# Novel Heat Shock Protein 90 Inhibitors Suppress P-Glycoprotein Activity and Overcome Multidrug Resistance in Cancer Cells

**DOI:** 10.3390/ijms20184575

**Published:** 2019-09-16

**Authors:** Jelena Dinić, Ana Podolski-Renić, Mirna Jovanović, Loana Musso, Ivanka Tsakovska, Ilza Pajeva, Sabrina Dallavalle, Milica Pešić

**Affiliations:** 1 Department of Neurobiology, Institute for Biological Research “Siniša Stanković”, University of Belgrade, Bulevar Despota Stefana 142, 11060 Belgrade, Serbia; jelena.dinic@ibiss.bg.ac.rs (J.D.); ana.podolski@ibiss.bg.ac.rs (A.P.-R.); mirna.jovanovic@ibiss.bg.ac.rs (M.J.); 2 Department of Food, Environmental and Nutritional Sciences, Università degli Studi di Milano, via Celoria 2, 20133 Milano, Italy; loana.musso@unimi.it (L.M.); sabrina.dallavalle@unimi.it (S.D.); 3 Department of QSAR and Molecular Modelling, Institute of Biophysics and Biomedical Engineering, Bulgarian Academy of Sciences, Acad. G. Bonchev Str., bl. 105, 1113 Sofia, Bulgaria; itsakovska@biomed.bas.bg (I.T.); pajeva@biomed.bas.bg (I.P.)

**Keywords:** isoxazolonaphthoquinones, Hsp90 inhibitors, P-glycoprotein inhibitors, cancer, multidrug resistance

## Abstract

Heat Shock Protein 90 (Hsp90) chaperone interacts with a broad range of client proteins involved in cancerogenesis and cancer progression. However, Hsp90 inhibitors were unsuccessful as anticancer agents due to their high toxicity, lack of selectivity against cancer cells and extrusion by membrane transporters responsible for multidrug resistance (MDR) such as P-glycoprotein (P-gp). Recognizing the potential of new compounds to inhibit P-gp function and/or expression is essential in the search for effective anticancer drugs. Eleven Hsp90 inhibitors containing an isoxazolonaphtoquinone core were synthesized and evaluated in two MDR models comprised of sensitive and corresponding resistant cancer cells with P-gp overexpression (human non-small cell lung carcinoma and colorectal adenocarcinoma). We investigated the effect of Hsp90 inhibitors on cell growth inhibition, P-gp activity and P-gp expression. Structure–activity relationship analysis was performed in respect to cell growth and P-gp inhibition. Compounds 5, 7, and 9 directly interacted with P-gp and inhibited its ATPase activity. Their potential P-gp binding site was identified by molecular docking studies. In addition, these compounds downregulated P-gp expression in MDR colorectal carcinoma cells, showed good relative selectivity towards cancer cells, while compound 5 reversed resistance to doxorubicin and paclitaxel in concentration-dependent manner. Therefore, compounds 5, 7 and 9 could be promising candidates for treating cancers with P-gp overexpression.

## 1. Introduction

Cancer is the second leading cause of death in the world, contributing to 8.8 million deaths in 2015 according to World Health Organization statistics [1]. Implementation of targeted therapeutics has provided hope for more efficient cancer treatment. High proliferative capacity of cancer cells presented a base for the selectivity of DNA damaging agents, also known as classic chemotherapeutics. Although still widely used in clinical practice, the application of this type of therapeutics is connected with severe damage in normal cells and systemic toxicity. New experimental tools and methodologies enabled the discovery of new biomarkers that could serve as targets for specific cancer types. The expected outcome of new therapeutic strategies is directed towards the inhibition of cancer cell growth and suppression of metastasis. However, the same obstacles recognized with classic chemotherapeutics continue to obstruct the efficacy of new targeted therapies.

Molecular chaperones play an essential role in almost all cellular events by assisting unfolded or misfolded macromolecules [2,3]. In addition, molecular chaperones are involved in soluble protein transport and subcellular localization of signaling intermediates. They also modulate transcription by epigenetic regulation of gene expression and chromatin modification. Heat shock proteins (HSPs) are key molecular chaperones whose expression is triggered in response to environmental stress conditions [4]. Cancer cells are more dependent on stress-related proteins including HSPs when compared to normal cells, since they need to rewire their signal transduction and metabolic pathways [5]. The most extensively studied target for cancer therapy is Hsp90, a heat shock protein linked to cancer cell proliferation, differentiation, invasion, metastasis, and drug resistance [6]. Hsp90 interacts with hundreds of client proteins and other signaling molecules including various oncogenes, p53, Akt, tyrosine kinases, steroid receptors, cytoskeletal proteins, importins, nucleoporins, and histones [7]. Hsp90 inhibitors based on the purine scaffold of ATP, the resorcinol scaffold of radicicol, the benzoquinone ansamycin scaffold of geldanamycin, and other chemical scaffolds shown to inhibit Hsp90 have been extensively studied both in vitro and as a part of clinical trials [8,9]. Despite certain promising results, issues such as toxicity, poor solubility, and limited bioavailability were responsible for the discontinuation of clinical development and lack of regulatory approval of Hsp90 inhibitors [7].

Significant limits for the efficient cancer treatment include the heterogeneity of cancer cells, development of resistance and evolutionary concept of regrowth that involves cancer stem cells or drug tolerant cells [10]. Resistance mechanisms in targeted therapy include activation of the downstream signaling molecules independently from the target, changes in the target molecule such as mutations or expression level, activation of the target by alternative mechanisms and drug elimination through export pumps—ATP Binding Cassette (ABC) transporters [11,12]. The most relevant and most studied representative of ABC membrane transporters involved in the classic mechanism of multidrug resistance (MDR) is P-glycoprotein (P-gp/MDR1/ABCB1). Its activity disables intracellular accumulation of chemotherapeutics and drug-target interaction [13]. Unfortunately, numerous anticancer therapeutics, including a variety of Hsp90 inhibitors [14], are substrates for P-gp. Nevertheless, some of the anticancer agents could be recognized as P-gp inhibitors. There is no final list of P-gp substrates and inhibitors; therefore, studying the efficacy of targeted therapeutics in cancer models with overexpression of P-gp constitutes a rational approach in preclinical studies.

Our hypothesis is that Hsp90 inhibitors of specific structure can directly or indirectly interact with P-gp and consequently modulate MDR. Directly, they may competitively inhibit P-gp behaving as P-gp substrates or may act as P-gp inhibitors. Indirectly, Hsp90 inhibitors may change the expression and activity of their client proteins involved in the regulation of P-gp expression. We previously reported a series of 3-aryl-naphtho[2,3-d]isoxazole-4,9-diones as inhibitors of Hsp90 [15]. Reaction of naphthoquinone (1) with suitable oximes (2) resulted in the generation of compounds 3–10, while regioselective 1,3-dipolar cycloaddition on the bromonaphthoquinones (12) produced compounds 13–15.

In this paper, we report our efforts to develop dual targeting molecules, with potential to act against both deregulated cancer metabolism by Hsp90 inhibition and MDR mechanism by P-gp inhibition. Eleven Hsp90 inhibitors (compounds 3–10, 13, 14 and 15) were analyzed to identify those more selective towards cancer cells with the ability to modulate MDR through interaction with P-gp. Docking studies were performed to identify the potential P-gp binding site of selected inhibitors. The anticancer activity of the compounds was tested on two MDR models comprised of P-gp overexpressing cancer cell lines established after continuous exposure to paclitaxel (PTX) or doxorubicin (DOX) [16,17] and their corresponding sensitive counterparts.

## 2. Results and Discussion

### 2.1. Structure–Activity Relationship Analysis in Relation to Cell Growth Inhibition of Cancer and Normal Cells

Quinones are a class of organic compounds that possess a range of biological activities, mainly associated with their redox properties. Numerous natural and synthetic quinones have been shown to display significant anticancer activity [18,19,20].

**Scheme 1:**
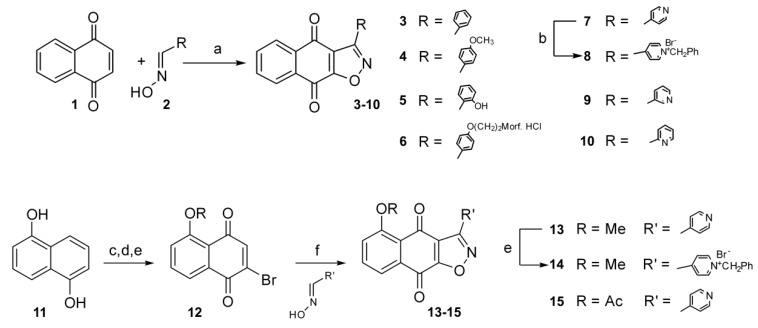
Synthesis of 3-aryl-naphtho[2,3-d]isoxazole-4,9-diones. Reagents and conditions: (a) TEA, 15% NaClO, CH_2_Cl_2_, 0 °C, 1.5 h; for 5: (a), then, BBr_3_, CH_2_Cl_2_, 0 °C, 1 h, rt, 30 min; (b) benzyl bromide, DMSO, rt, overnight; (c) Ac_2_O Py, rt, 3 h; (d) NBS, AcOH/H_2_O, 50 °C, 2 h; (e) for 13: (i) H_2_SO_4_ 1.5 M, EtOH, reflux, 3 h; (ii) Mel, Ag_2_O, CH_2_Cl_2_, rt, overnight; (f) TEA, 15% NaClO, CH_2_Cl_2_, 0 °C, 1.5 h; (e) benzyl bromide, DMSO, rt, overnight.

**Figure 1:**
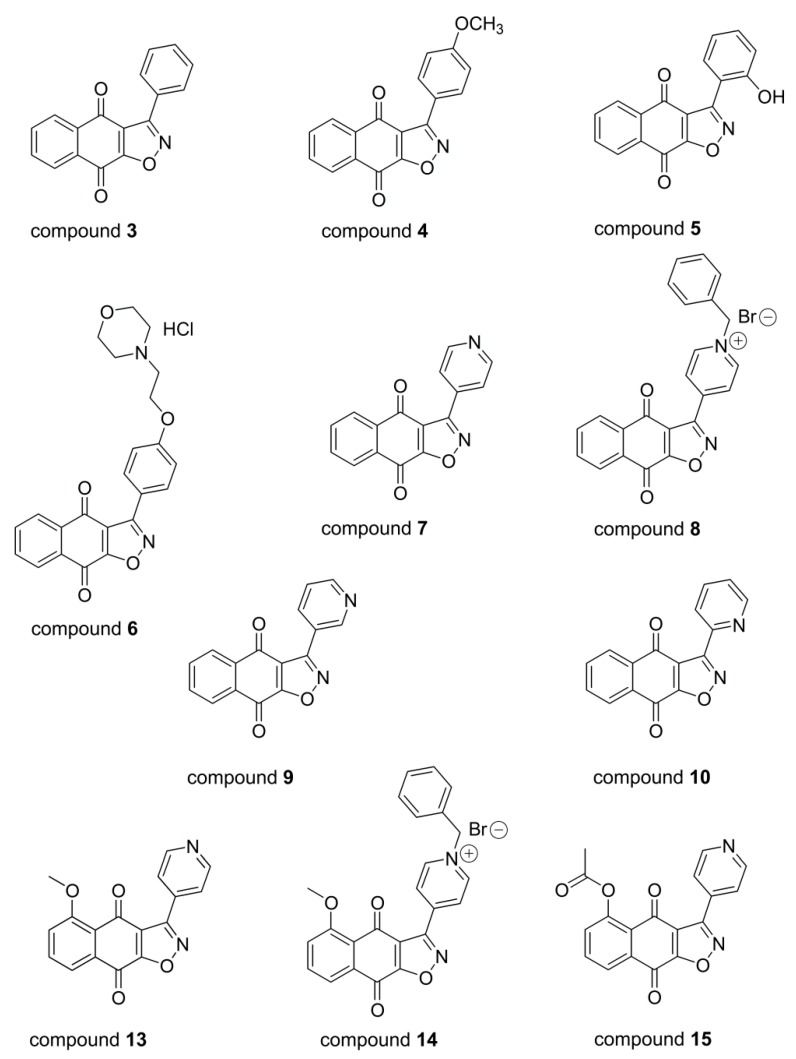
Chemical structures of Hsp90 inhibitors.

A series of 3-aryl-naphtho[2,3-d]isoxazole-4,9-diones were synthesized (Scheme 1, Figure 1) and their growth inhibition activity was tested in vitro on human non-small cell lung carcinoma NCI-H460, colorectal carcinoma DLD1 and their corresponding MDR cells (NCI-H460/R and DLD1-TxR, respectively), as well as normal embryonic lung fibroblasts MRC-5. The differences in cell growth inhibition between cancer and normal cells obtained by the MTT assay after 72 h treatment are presented in Table 1. The effect of compounds is presented as IC_50_ values that correspond to the 50% of cell growth inhibition.

Isoxazolo-fused naphthoquinone 3 triggered a considerable growth inhibition in investigated cancer cell lines (IC_50_ = 785 nM for NCI-H460; IC_50_ = 925 nM for DLD1), and, interestingly, showed better efficacy in cells with MDR phenotype. A phenomenon in which development of MDR phenotype in cancer cells confers higher sensitivity to drugs compared to parental cell is termed collateral sensitivity [21]. The introduction of a polar pOMe group on the phenyl ring in compound 4 caused a slight reduction in the activity (IC_50_ = 935 nM for NCI-H460; IC_50_ = 1523 nM for DLD1), but the efficacy on both MDR cancer cells remained higher than in the corresponding sensitive cells. The activity was further reduced by the introduction of a hydroxy group in the ortho position on the phenyl ring. Consequently, compound 5 had lower activity in both cell lines (IC_50_ = 5722 nM for NCI-H460; IC_50_ = 5683 nM for DLD1). The effect was further compromised in the presence of MDR phenotype.

As the introduction of substituents at the ortho position on the benzyl ring seemed to be less tolerated than in para position (compound 5 vs. compound 4), we intended to investigate the effect of the introduction of other polar moieties at the para position, with the aim of increasing the polarity and possibly the water solubility of the compounds. Derivative 6, containing a hydrophilic morpholine hydrochloride moiety, showed strong activity in investigated cancer cell lines (IC_50_ = 807 nM for NCI-H460; IC_50_ = 439 nM for DLD1). Interestingly, the compound evaded resistance in DLD1-TxR cells and showed collateral sensitivity in NCI-H460/R cells. It is important to point out that all isoxazole-containing compounds with highest activity described in the literature contain morpholine moiety [22,23,24].

Substitution of the phenyl ring with a pyridine moiety led to an increase in activity, as compound 7 displayed a very strong effect (IC_50_ = 199 nM for NCI-H460; IC_50_ = 156 nM for DLD1). The compound evaded resistance in both MDR cancer cell lines and exhibited higher activity than the corresponding phenyl substituted compound 3.

Shifting the position of the nitrogen atom to position 3 conserved a strong activity (compound 9, IC_50_ = 96 nM for NCI-H460; IC_50_ = 45 nM for DLD1), whereas the presence of the nitrogen atom in position 2 had an unfavorable effect (compound 10, IC_50_ = 1269 nM for NCI-H460; IC_50_ = 2645 nM for DLD1). This trend was in line with the result found for compound 5, having a polar group on the ortho position of the aromatic ring. Regardless of reduced activity, compound 10 overcame resistance in NCI-H460/R and displayed collateral sensitivity in DLD1-TxR cells.

The introduction of an acetoxy group on the naphtoquinone ring (compound 15) further increased the activity on sensitive cancer cell lines (IC_50_ = 52 nM for NCI-H460; IC_50_ = 44 nM for DLD1). The activity was maintained in resistant NCI-H460/R cells (IC_50_ = 39 nM), whereas it was lower in DLD1-TxR cell line (IC_50_ = 193 nM). The presence of a methoxy group in the same position (compound 13) caused a drop in the activity (IC_50_ = 1895 nM for DLD1; IC_50_ = 1250 nM for NCI-H460). Nevertheless, compound 13 evaded resistance mechanisms in NCI-H460/R and demonstrated collateral sensitivity in DLD1-TxR cell line.

To increase the polarity and the water solubility, the pyridine derivatives 7 and 13 were alkylated with benzyl bromide, to give the corresponding quaternary salts 8 and 14 (Scheme 1). This modification was disadvantageous for the activity of compound 14 in DLD1 cell line (IC_50_ = 7449 nM for DLD1; IC_50_ = 978 nM for NCI-H460) and the effect was further compromised in the presence of MDR phenotype in both cell lines. On the contrary, alkylation of compound 7 did not cause a reduction in the activity in sensitive cancer cell lines (compound 8, IC_50_ = 159 nM for NCI-H460; IC_50_ = 251 nM for DLD1), but this effect was also compromised in the presence of the MDR phenotype.

Compared to normal MRC-5 cell line, eight out of eleven investigated compounds (4–9, 14 and 15) displayed relative selectivity towards cancer cells.

### 2.2. Comparison of Hsp90 Inhibitors’ Cell Growth Inhibition with HSP90 mRNA Expression Profile in Cancer Cells and Hsp90 Affinity Binding

The expression of HSP90 was analyzed from total RNA samples of NCI-H460, NCI-H460/R, DLD1, DLD1-TxR cells by qPCR (Table 2). The expression of HSP90 on mRNA level was affected by the development of MDR phenotype in both resistant cell lines. In NCI-H460/R cells, mRNA HSP90 expression decreased approximately four-fold compared to sensitive NCI-H460 cells. In contrast, the expression of HSP90 was reduced by 25% in DLD1-TxR cells compared to their parental cell line.

**Figure 2:**
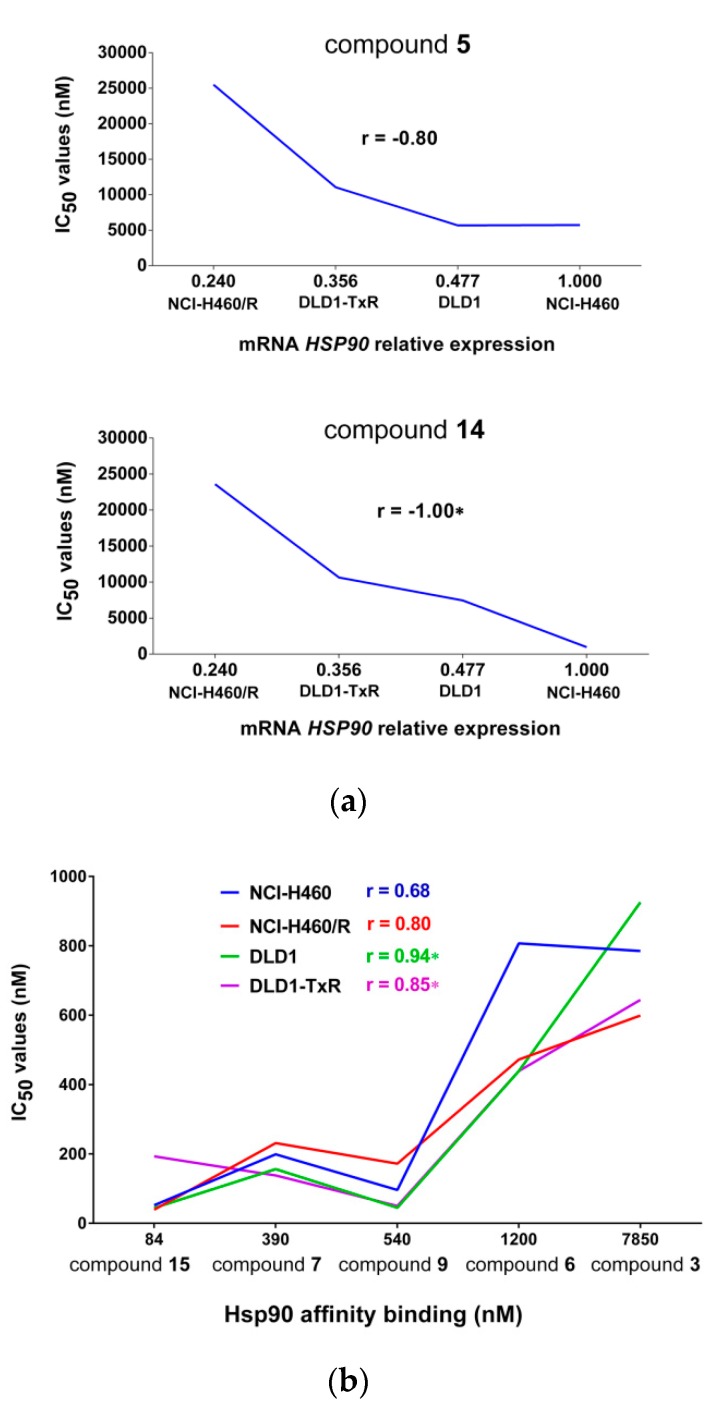
Cell growth inhibition potential of Hsp90 inhibitors correlates with the level of Hsp90 expression and Hsp90 affinity binding. (**a**) Negative correlation between IC_50_ values and mRNA *HSP90* relative expression. Spearman correlation indicates that the effect of compounds 5 and 14 on growth inhibition is stronger in cell lines with higher mRNA *HSP90* expression (*r* = Spearman’s correlation coefficient). Statistical significance: *p* < 0.05 (*) (**b**) Positive correlation between Hsp90 inhibitors’ effect on cell growth inhibition and Hsp90 affinity binding. Pearson correlation is applicable only for Hsp90 inhibitors with strong effect on cell growth (IC_50_ < 1000 nM). (*r* = Pearson’s correlation coefficient). Statistical significance: *p* < 0.05 (*).

The obtained IC_50_ values from Table 1 were used to evaluate the influence of *HSP90* mRNA expression level on the cell growth inhibition by Hsp90 inhibitors (Figure 2a). Spearman correlation indicates that the *HSP90* mRNA expression profile of cell lines affected the cell growth inhibitory potential of only two inhibitors, compounds 5 and 14 (*r* < −0.5). The decreased expression of *HSP90* in MDR cell lines was accompanied by resistance to these Hsp90 inhibitors. The greater difference in *HSP90* expression between NCI-H460 and NCI-H460/R cells, also resulted in greater difference in their effect, compared to the other sensitive/resistant pair of cells.

When the IC_50_ values obtained by the MTT assay were compared to Hsp90 affinity binding IC_50_ values (Table 1), a positive Pearson correlation (*r* > 0.5) was found for all cancer cell lines (Figure 2b). However, this correlation is applicable only to Hsp90 inhibitors with IC_50_ values < 1000 nM (compounds 3, 6, 7, 9, and 15). Neither positive nor negative correlations were found for compounds 4, 5, 8, 10, 13 and 14 with IC_50_ values ≥ 1000 nM. This finding indicates that compounds with higher Hsp90 binding affinity also possess a stronger cell growth inhibitory potential.

### 2.3. Hsp90 Inhibitors Influence P-gp Activity and Expression

P-gp, as a member of the ATP-binding cassette transporter family, acts as an efflux pump for a variety of anticancer agents [25,26,27]. The efficacy of Hsp90 inhibitors as anticancer agents has been previously linked to P-gp expression and MDR phenotype [28]. Resistance to Hsp90 inhibitors such as benzoquinone ansamycins GdA and herbimycin A was observed in doxorubicin-selected human breast cancer MCF7/ADRR cells and associated with the overexpression of P-gp [29]. Another Hsp90 inhibitor, 17-AAG, was reported to be less effective in cells overexpressing efflux pumps [28,30]. On the contrary, synthetic purine- and pyrazole-based inhibitors of Hsp90, which are not P-gp substrates, evade the resistance mechanisms in MDR cancer cells [31,32,33]. As some of our Hsp90 inhibitors evaded resistance in both MDR cancer cell lines (Table 1), we next analyzed their interaction with the P-gp transporter.

**Figure 3:**
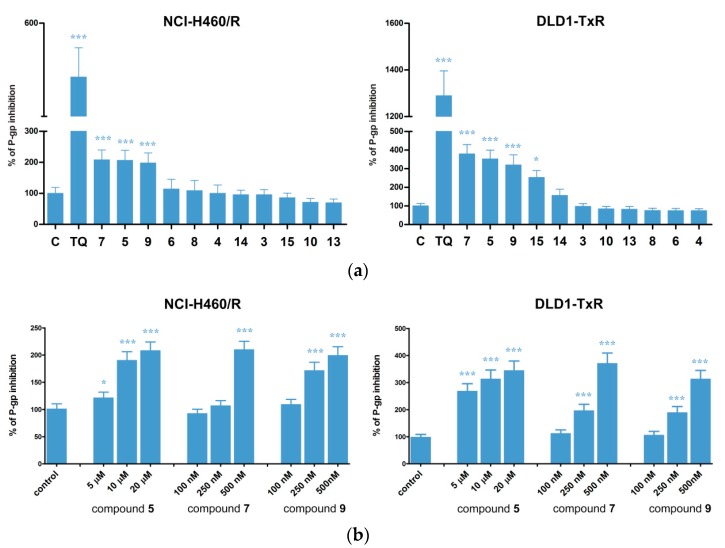
Suppression of P-gp activity induced by Hsp90 inhibitors. (**a**) The inhibition of P-gp activity in MDR NCI-H460/R and DLD1-TxR cells after 30 min treatment with Hsp90 inhibitors. (**b**) Dose dependent effects on P-gp inhibition in NCI-H460/R and DLD1-TxR cells after 30 min treatment with Hsp90 inhibitors. Tariquidar (TQ) is included as positive control. The results are expressed as mean ± SD. Statistical significance to untreated control from three independent experiments: *p* < 0.05 (*), *p* < 0.001 (***).

To study the effect of Hsp90 inhibitors on P-gp activity in MDR cancer cells, intracellular accumulation of the P-gp substrate rhodamine 123 (Rho 123) was analyzed by flow cytometry after 30 min treatment (Figure 3a). A well-known inhibitor of P-gp activity, tariquidar (TQ), was included as a positive control.

Compound 3, with a phenyl group on the isoxazolo-fused naphthoquinone ring, did not influence the P-gp activity in either of the MDR cell lines (Figure 3a). The introduction of polar p-methoxy or morpholino groups (compounds 4 and 6) did not have any positive effects, whereas the presence of an o-hydroxy group (compound 5) had a significant beneficial effect on P-gp inhibition.

Compounds 9 and 7, both containing a pyridinyl moiety on the isoxazolo-fused naphthoquinone ring, also showed an evident increase in Rho 123 accumulation.

Conversely, a different linkage of the pyridinyl moiety (pyridin-2-yl vs. pyridin-4-yl), caused a reduction in activity (compound 10 vs. compound 7). The activity was also decreased with the introduction of a methoxy group in position 5 of compound 7 (compound 13 vs. compound 7), whereas the introduction of an acetoxy moiety in the same position gave a compound that maintained a certain activity but only in DLD1-TxR cells (compound 15).

The alkylation on the pyridine nitrogen by a benzyl group to obtain the corresponding pyridium salt 8 was detrimental. The activity was partially restored with the introduction of a methoxy group in position 5 (compound 14), as observed in DLD1-TxR cell line (Figure 3a).

We tried to rationalize the obtained results in light of physicochemical properties of this small collection of compounds. In this respect the most active compounds (5, 9, and 7) have logP > 2 (Table 1). Increasing the hydrophilicity appeared to have a negative effect on the compound activity. Indeed, compound 8, with a morfolino hydrochloride moiety (see compounds 8 vs. 7) showed modest activity. However, we cannot exclude that the reduced activity is due to the increased Van der Walls volume of the compounds due to the introduction of bulky groups. In fact, the same trend is not followed for compounds 13 and 14.

The presence of a methoxy group in position 5 seems to be unfavorable (compound 13 vs. compound 7), as well as a methoxy group at the para position of the phenyl group (compound 4 vs. compound 3). Conversely, the hydroxyl group on the phenyl ring in compound 5 has a significant effect on the P-gp inhibition (compound 5 vs. compound 3).

Moreover, in the series containing the pyridinyl group in different positions (compound 9: pyridin-3-yl; compound 7: pyridin-4-yl; compound 10: pyridin-2-yl) the reduction of pKa (2.40 for compound 10, compared to 4.18 and 3.76 for compounds 9 and 7) led to a drop in activity.

Compounds 5, 7 and 9 which demonstrated the most prominent effect in both MDR cell lines, were selected for a detailed investigation regarding concentration dependent inhibition of the P-gp activity. Indeed, all three compounds exerted concentration dependent effect, as indicated by the increase in rhodamine 123 accumulation (Figure 3b).

The main function of Hsp90, as a highly conserved molecular chaperone, is to regulate folding, stability, and the activity of numerous Hsp90-associated proteins [34]. The ERK pathway is one of the most important cell growth signaling pathways in cancer and its components are involved in the regulation of P-gp expression in cancer cells [35,36,37]. Hsp90 enables the kinase activity of the ERK pathway proteins such as Raf and MEK by regulating their folding which suggests that the application of Hsp90 inhibitors could block the ERK signaling pathway, downregulating P-gp expression as a result. Colorectal cancer has a high incidence of *KRAS* mutations and the constitutive activation of Ras/Raf/MEK/ERK signaling [38]. This makes this type of cancer exceptionally interesting regarding Hsp90 inhibition due to sensitivity of these signal transduction pathways to Hsp90 inhibitors [39,40,41,42]. The effectiveness of targeting Hsp90 in human colon cancer has been shown both in vitro and in vivo [30,33,42,43].

**Figure 4:**
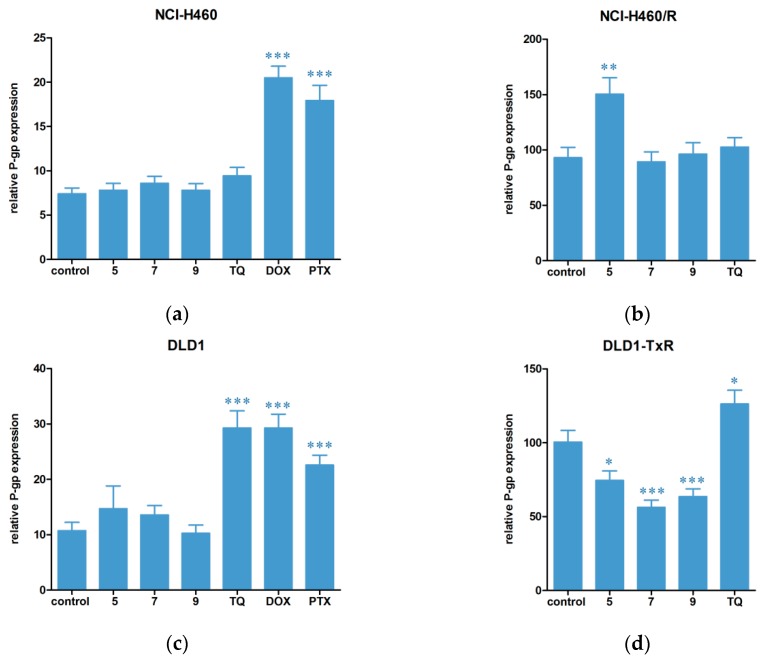
Changes in P-gp expression at protein level after treatment with Hsp90 inhibitors. Changes in P-gp expression in (**a**) parental NCI-H460 cell line, (**b**) MDR NCI-H460/R cell line, (**c**) parental DLD1 cell line and (**d**) MDR DLD1-TxR cell line after 72 h treatment with compounds 5, 7 and 9. Doxorubicin DOX, paclitaxel (PTX) and tariquidar (TQ) are included as positive controls. The results are expressed as mean ± SEM. Statistical significance to untreated control from three independent experiments: *p* < 0.05 (*), *p* < 0.01 (**), *p* < 0.001 (***).

Accordingly, we next analyzed the influence of our Hsp90 inhibitors (compounds 5, 7 and 9) on P-gp expression in parental (sensitive) and MDR non-small cell lung carcinoma and colorectal adenocarcinoma cells (Figure 4). Important findings were obtained on both sensitive cell lines NCI-H460 and DLD1 (Figure 4a,c). Namely, compounds 5, 7 and 9 did not change P-gp expression in sensitive cancer cells quite oppositely from classic chemotherapeutics DOX and PTX that significantly increased P-gp expression. Interestingly, TQ did not influence the P-gp expression in NCI-H460 and NCI-H460/R cells (Figure 4a,b), while increased P-gp expression in DLD1 and DLD1-TxR cells was observed after TQ treatment (Figure 4c,d). Compound 5 significantly increased P-gp expression only in NCI-H460/R cells (Figure 4b) and this could be attributed to the compensatory mechanism due to compound 5’s strong activity against P-gp functioning. Importantly, all tested compounds significantly decreased P-gp expression in DLD1-TxR cell line (Figure 4d).

**Figure 5:**
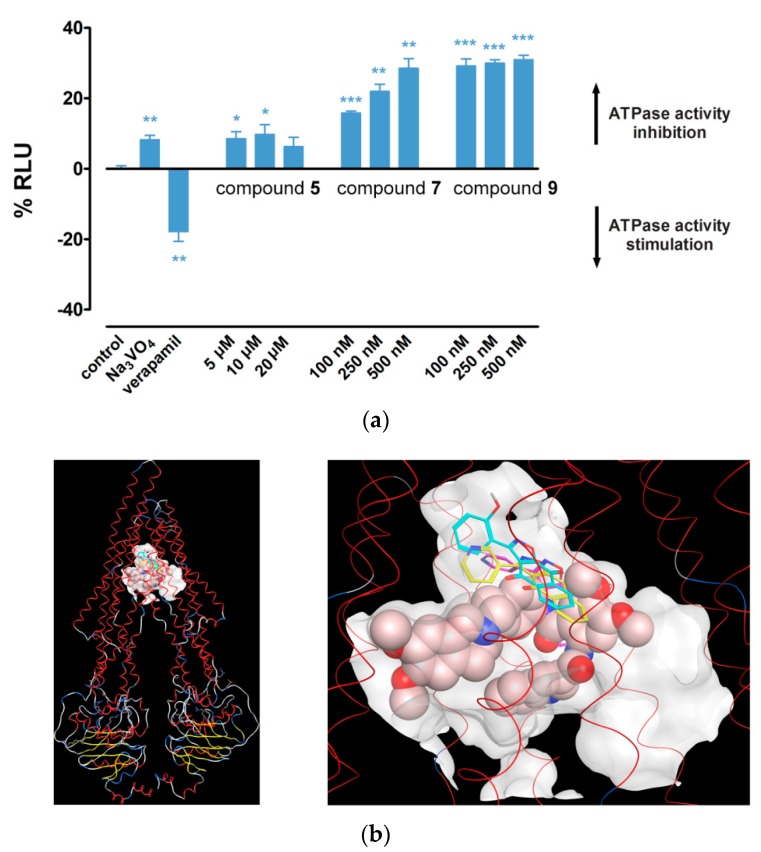
Hsp90 inhibitors affect the ATPase activity of P-gp by direct binding in the P-gp cavity. (**a**) Changes in P-gp ATPase activity after treatment with Hsp90 inhibitors. The y-axis represents percentage of change in relative luminescence units (RLU). Control is normalized to zero. Na_3_VO_4_ and verapamil are included as positive controls. The results are expressed as mean ± SEM. Statistical significance: *p* < 0.05 (*), *p* < 0.01 (**), *p* < 0.001 (***). (**b**) Binding poses of compound 5 (C atoms in cyan), compound 7 (C atoms in magenta), compound 9 (C atoms in yellow), and the positive control tariquidar (space filled atoms representation) in the P-gp cavity. The protein backbone is rendered as a line ribbon, and the helices are colored red. The binding site is outlined by the molecular surfaces of the X-ray structures of taxol and two zosuquidar molecules. Both general (left) and close-up views (right) of the binding site are presented.

In addition to a prominent effect on P-gp function, compounds 5, 7 and 9 exhibited strongest selectivity towards cancer cells as detected by the MTT assay. For this reason, compounds 5, 7 and 9 were selected to evaluate their ability to inhibit the ATPase activity of P-gp transporter. Verapamil, a P-gp substrate and competitive inhibitor, and sodium orthovanadate (Na_3_VO_4_), a noncompetitive inhibitor of P-gp ATPase activity, were used as positive controls. The results are reported in Figure 5a. Compound 7 showed a potent dose-dependent inhibition. Compound 9 showed a significant inhibition as well; however, it seemed to be independent from the concentration. The smallest effect on ATPase activity was observed with compound 5.

### 2.4. Hsp90 Inhibitors have Potential to Modulate P-gp Activity via Direct Binding

*In silico* studies were further undertaken to elucidate possible interactions of compounds 5, 7 and 9 with P-gp. In particular, docking simulations were performed using two structures of P-gp extracted from Protein Data Bank (PDB, www.rcsb.org)—human taxol-bound P-gp (PDB ID 6QEX) and human-mouse chimeric zosuquidar-bound P-gp (PDB ID 6QEE, two molecules of zosuquidar co-crystalized) [44]. These structures have been recently resolved and provide an excellent opportunity for comparison of substrate- and inhibitor-bound structures in the drug-binding cavity of P-gp. Particularly, it has been noted that the inhibited P-gp structure reveals two molecules of zosuquidar that occupy the same central occluded pocket where the taxol is bound in [45].

Based on this observation, the binding site for docking simulations in both structures was defined by superposition of the X-ray protein structures and identification of the space positions of the three ligands (taxol and two zosuquidars) that fit the cavity where the docking poses to be placed. Similar docking results were produced in both structures and finally the taxol-bound P-gp structure was used in the simulations.

Different docking protocols were compared and the one with the best performance on re-docking of the X-ray taxol and zosuquidar structures was selected in terms of (i) similarity between the generated poses and the corresponding structures in the crystal complex, and (ii) calculated scores that approximate the binding affinity (lower scores indicate more favorable interactions).

The compounds 5, 7 and 9, as well as TQ (used as a reference compound in the Rho 123 accumulation assay), were docked. The docking results confirmed the potential of the investigated compounds to bind in the P-gp cavity. The range of the scores for the first five poses of compounds 5, 7 and 9 and TQ were (−9.49 ÷ −741), (−8.54 ÷ −7.86), (−9.78 ÷ −8.23) and (−16.41 ÷ −13.66), respectively. For comparison, the scores of the re-docked X-ray ligands were in the range (−15.62 ÷ −13.87) for taxol and (−13.17 ÷ −10.84) for zosuquidars. The docking results are in agreement with the in vitro results on Rho 123 accumulation, where the effects of the three studied compounds are comparable and lower than the effect of the positive control TQ (Figure 3). The selected binding poses of the three Hsp90 inhibitors and TQ resulting from the performed docking are presented in Figure 5b. As seen from the figure, the investigated compounds occupy the same region within the cavity defined by the X-ray ligands. At the same time, they are positioned closer to the upper site (near the outside of the cell) compared to tariquidar. This is reasonable, taking into consideration that the structures under investigations are smaller in size than TQ and binding into a more closed and burred site could better stabilize their position.

The analysis of the ligand-protein interactions of the selected poses outlines a number of specific interactions that may potentially occur upon binding, including hydrogen bonding with Gln725 and arene–arene interactions with Phe336 for compound 7, as well as hydrogen bonding with Met 986, arene–arene interactions with Phe983 and arene–H interactions with Phe72 for compound 5. No specific interactions were recorded for compound 9.

We previously identified a binding site for Rho 123 (R-site) in the binding pocket of the P-gp protein cavity [46] that involved the residues Ile340, Leu975, Val981, and Val982, proposed by Loo and Clarke for the binding site of rhodamines in experiments with thiol-reactive rhodamine derivatives [47]. It is worth noting that the residues involved in the binding sites of the studied compounds include those shown to form the R-site, and those performing specific interactions and being in the closest contact to Rho 123 (Met68, Met69, Phe72, Phe336, Tyr953). Interestingly, the site of compound 7 includes also the residue Ile340 experimentally proven to relate to the R-site [47].

In a later study, we also proposed binding sites for the P-gp inhibitors tariquidar and elacridar [48]. In the current study, the highly ranked poses of TQ involved residues that overlapped with those previously identified, including hydrogen bonding with Gln990. In agreement, the binding sites for compounds 5, 7 and 9 proposed here involved residues that also shaped the binding site of TQ, as illustrated by overlapping of the compounds and TQ (Figure 5b). Based on these results, and in accordance with the experimental findings (Figure 3), we can suggest that the binding sites of compounds 5, 7 and 9 may partially overlap with the R-site, implying that these compounds may act as competitive inhibitors of this P-gp substrate.

### 2.5. Hsp90 Inhibitors Sensitizes MDR Cancer Cells to DOX and PTX

**Figure 6:**
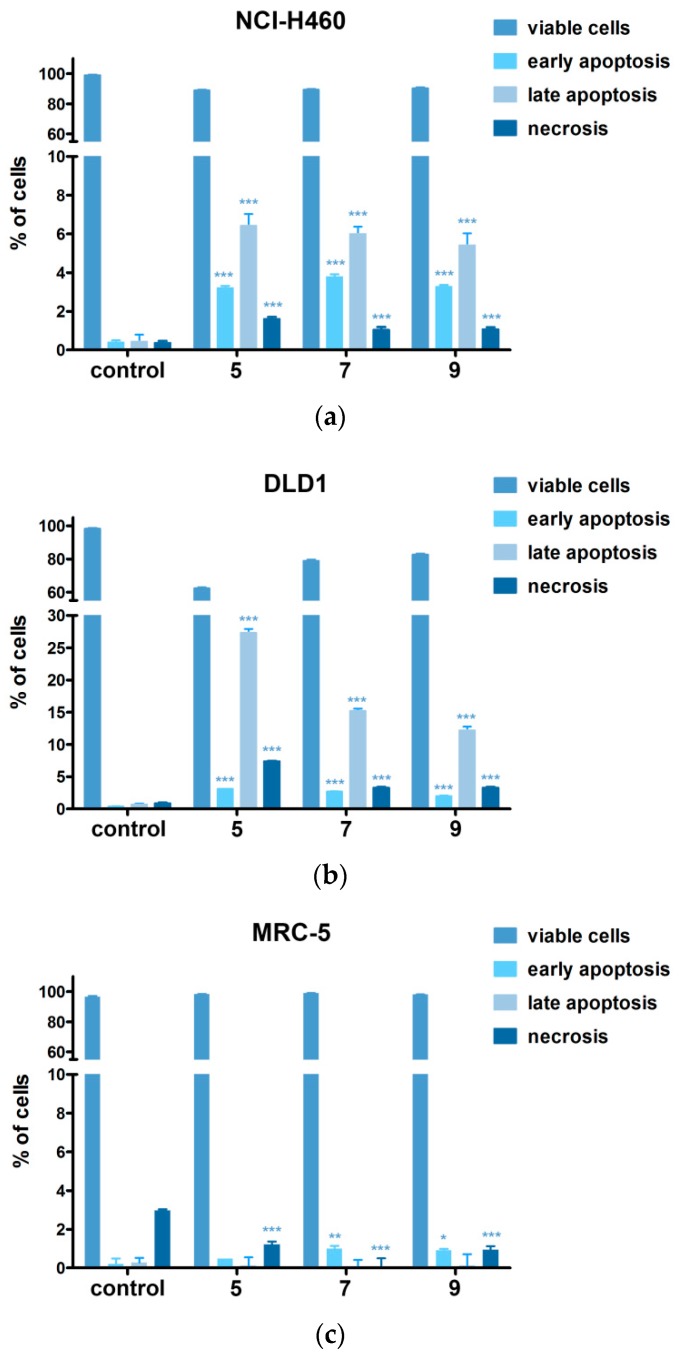
Cell death analysis confirmed good selectivity profile of Hsp90 inhibitors. Cell death induction in (**a**) NCI-H460, (**b**) DLD1 and (**c**) MRC-5 cells assessed by AV/PI staining after 72 h treatment with compounds 5, 7 and 9. Flow cytometric assay distinguishes viable (AV−PI−), early apoptotic (AV+PI−), late apoptotic (AV+PI+) and necrotic (AV−PI+) cells. The results are expressed as mean ± SEM. Statistical significance to untreated control from three experiments: *p* < 0.05 (*), *p* < 0.01 (**), *p* < 0.001 (***).

Due to the possible induction of the sever cytotoxicity when Hsp90 inhibitors are applied with classic chemotherapeutics DOX and PTX, we analyzed and compared the induction and type of cell death between cancer and normal cells exposed to compounds 5, 7 and 9 (Figure 6). The results showed that all compounds induced cell death, early and late apoptosis, in cancer cells NCI-H460 and DLD1 (Figure 6a,b). The application of Hsp90 inhibitors led to the significant decrease in portion of necrotic cells observed in normal untreated MRC-5 cells (Figure 6c). By this means we further confirmed good selectivity profile of compounds 5, 7 and 9 and continued to test the combined strategy with Hsp90 inhibitors and classic chemotherapeutics DOX and PTX.

Considering that compound 5 showed the best selectivity index (10.64, Table 1), we tested the interaction between 5 and DOX as well as 5 and PTX during combined treatments in NCI-H460/R and DLD1-TxR cells, respectively.

**Figure 7:**
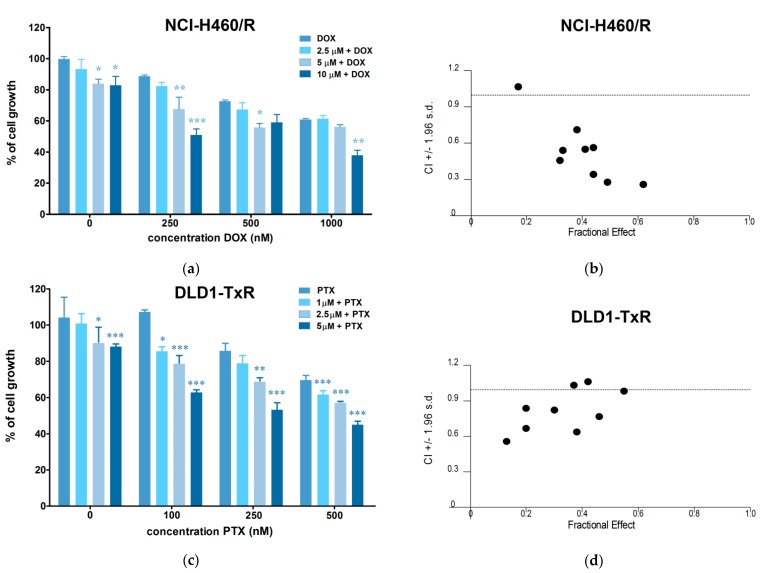
Hsp90 inhibitors compound 5 chemosensitizes MDR cancer cells to doxorubicin (DOX) and paclitaxel (PTX). (**a**) Cell growth inhibition by DOX in single and combination treatments with Hsp90 inhibitors in MDR NCI-H460/R cells. (**b**) Synergistic type of interaction between 5 and DOX. (**c**) Cell growth inhibition by PTX in single and combination treatments with Hsp90 inhibitors in MDR DLD1-TxR cells. (**d**) Synergistic type of interaction between 5 and PTX. The results are expressed as mean ± SEM. Statistical significance to DOX or PTX alone from three experiments: *p* < 0.05 (*), *p* < 0.01 (**), *p* < 0.001 (***). CI values below 1 depict synergistic effect.

Compound 5 increased the sensitivity of both MDR cell lines to classic chemotherapeutics (Figure 7a,c) displaying synergistic interaction with DOX and PTX (Figure 7b,d). Decreased IC_50_ values for DOX and PTX in all tested combinations are summarized in Table 3. The effect of compound 5 on DOX resistance reversal increased in a concentration-dependent manner ranging from 1.31-fold to 5.28-fold and, similarly, PTX resistance was reversed by 1.80-fold to 5.27-fold.

It is important to highlight that the chemosensitization of NCI-H460/R and DLD1-TxR cells was also achieved by concentrations considerably lower than those required for a significant cell growth inhibition or P-gp inhibition. Consequently, application of compound 5 in non-toxic concentrations as low as 1 and 2.5 µM, has the potential to increase intracellular accumulation of chemotherapeutics by inhibiting the P-gp efflux in cells with MDR phenotype.

Analysis of the nature of the interaction between the two drugs revealed that some combinations of compound 5 with DOX and PTX have a pronounced synergistic effect with most Combination Index (CI) values below 1 (Figure 7b,d). The combination of drugs with different modes of action is an efficient strategy in chemotherapeutic cancer treatment, as it overcomes drug resistance and reduces adverse side effects by decreasing drug dosage based on their synergy. Therefore, Hsp90 inhibitors could offer a promising approach for the treatment of resistant cancers aimed at reducing therapy resistance based on efflux pump overexpression.

## 3. Materials and Methods

### 3.1. Drugs

Hsp90 inhibitors (compounds 3–10, and 13, 14, and 15) containing an isoxazolonaphtoquinone core were dissolved in dimethyl sulfoxide (DMSO) as 20 mM aliquots and kept at room temperature until use. Tariquidar (Avaant Pharmaceuticals, London, UK) was stored as 10 mM aliquots at −20 °C. Doxorubicin—DOX (EBEWE Arzneimittel GmbH, Vienna, Austria) was diluted in sterile water and stored as 1 mM aliquots at −20 °C. Paclitaxel—PTX (Sigma-Aldrich Chemie Gmbh, Hamburg, Germany) was diluted in absolute ethanol and 1 mM aliquots were stored at −20 °C. Before treatment, all drugs were freshly diluted in sterile water.

### 3.2. Reagents

RPMI 1640 medium, fetal bovine serum (FBS), antibiotic-antimycotic solution, penicilin-streptomicin solution, L-glutamine and trypsin/EDTA were purchased from Biological Industries (Beit Haemek, Israel). Rhodamine 123, DMSO and thiazolyl blue tetrazolium bromide (MTT) were purchased from Sigma-Aldrich Chemie GmbH (Hamburg, Germany). FITC-conjugated anti-P-glycoprotein antibody was obtained from BD Biosciences (Plymouth, UK). Annexin V-FITC apoptosis detection kit with Propidium Iodide was purchased from Abcam (Cambridge, UK).

### 3.3. Chemistry

The 1,3-dipolar cycloaddition of benzonitriloxides to quinones has been exploited for the synthesis of isoxazolo-fused naphthoquinones as previously described [15]. Thus, compounds 3–10 were prepared via reaction of naphthoquinone with suitable nitrile oxides obtained in situ by treating the corresponding oximes with triethylamine and aqueous NaClO in dichloromethane (Scheme 1). Compounds 13–15 were obtained by regioselective 1,3-dipolar cycloaddition on the bromonaphthoquinones 12, followed in the case of 14 by alkylation with benzyl bromide (Scheme 1).

Computer calculations of the logP and pKa values were performed by online software Chemicalize (chemicalize.com) developed by ChemAxon (Budapest, Hungary).

### 3.4. Cell Culture

Human colorectal carcinoma (DLD1), non-small cell lung carcinoma (NCI-H460), and normal embryonic lung fibroblast (MRC-5) cell lines were purchased from the American Type Culture Collection, Rockville, MD. P-gp overexpressing multidrug resistant DLD1-TxR cells were selected by continuous exposure to stepwise increasing concentrations of paclitaxel from DLD1 cells, while NCI-H460/R cells were selected from NCI-H460 cells after DOX selective pressure. MDR cancer cell lines, DLD1-TxR and NCI-H460/R, and their sensitive counterparts were maintained in RPMI 1640 medium supplemented with 10% FBS, 2 mM L-glutamine, and 10,000 U/mL penicillin, 10 mg/mL streptomycin, 25 mg/mL amphotericin B solutions. MRC-5 cells were cultured in DMEM supplemented with 10% FBS, 4 g/L glucose, 2 mM L-glutamine and 5000 U/mL penicillin, 5 mg/mL streptomycin solution. All cell lines were sub-cultured at 72 h intervals using 0.25% trypsin/EDTA and seeded into a fresh medium at 8000 cells/cm^2^. All cell lines were maintained at 37 °C in a humidified 5% CO_2_ atmosphere.

### 3.5. MTT Assay

Cell viability was assessed by the MTT assay (AppliChem GmbH, Darmstadt, Germany). Cells grown in 25 cm^2^ tissue flasks were trypsinized and 1000 cells/well were seeded into flat-bottomed 96-well tissue culture plates and incubated overnight. Subsequently, the cells were treated with increasing concentrations of Hsp90 inhibitors for 72 h: compound 3 (0.1–2.5 µM), compound 4 (0.25–5 µM), compound 5 (0.5–25 µM), compound 6 (50–1000 nM), compound 7 (10–250 nM), compound 8 (0.25–5 µM), compound 9 (10–250 nM), compound 10 (0.5–25 µM), compound 13 (0.5–10 µM), compound 14 (1–25 µM), and compound 15 (10–250 nM). Due to the fact that the concentration range of some compounds exceeded 10 µM (compound 5, 10 and 14), the same DMSO concentration (0.125%) as was reached in 25 µM treatments was applied to exclude the effect of DMSO in these treatments. No cytotoxicity was observed after the application of 0.125% DMSO in all tested cell lines. After treatment, MTT was added to each well in a final concentration of 0.2 mg/mL for 4 h. Formazan product was dissolved in 200 µl of DMSO, and the absorbance was measured at 540 nm using an automatic LKB 5060-006 Micro Plate Reader (Vienna, Austria). IC_50_ value was defined as a concentration of the drug that inhibited cell growth by 50% and was calculated by nonlinear regression analysis using GraphPad Prism 6.0 for Windows (La Jolla, CA, USA). Relative selectivity towards cancer cells was calculated as a relation between IC_50_ value obtained in MRC-5 cells and an average IC_50_ value from four tested cancer cell lines.

### 3.6. RNA Extraction and Reverse Transcription Reaction

Total RNA was isolated from untreated NCI-H460, NCI-H460/R, DLD1 and DLD1-TxR cells. The isolation was carried out using Trizol^®^ reagent (Invitrogen Life Technologies, Waltham, MA, USA) according to the manufacturer’s instructions. RNA was quantified by spectrophotometry and quality was determined by agarose gel electrophoresis. Reverse transcription reactions were performed using 2 µg of total RNA, with a high-capacity cDNA reverse transcription kit (Applied Biosystems, Waltham, MA, USA), following the manufacturer’s instructions.

### 3.7. Quantitative Real-Time PCR

Quantitative real-time PCR (qPCR) was used to evaluate *HSP90α* expression in NCI-H460, NCI-H460/R, DLD1 and DLD1-TxR cells. The reactions were performed using a Maxima SYBR Green/ROX qPCR Master Mix (Thermo Scientific, Waltham, MA, USA) on an ABI PRISM 7000 Sequence Detection System (Applied Biosystems, Waltham, MA, USA) according to the manufacturer’s recommendations, using 100 ng cDNA in conjunction with primers specific for *ABCB1* [49], *HSP90α* [50] and *ACTB* as internal control for normalization [51]. Each sample was tested in triplicate and relative gene expression levels were analyzed by the 2^−ΔΔCt^ method [52].

### 3.8. Flow Cytometric Analysis of P-gp Expression

P-glycoprotein expression level in NCI-H460, NCI-H460/R, DLD1, and DLD1-TxR and cells was measured by flow cytometry. All cell lines were seeded in adherent 6-well plates and treated with Hsp90 inhibitors for 72 h at the following concentrations: 5 µM compound 5, 200 nM compound 7, 100 nM compound 9 (NCI-H460 and DLD1), and 10 µM compound 5, 500 nM compound 7, 500 nM compound 9 (NCI-H460/R and DLD1-TxR). Cells were also treated with 10 nM TQ, a non-competitive P-gp inhibitor, which served as a positive control. In addition, sensitive cells (NCI-H460 and DLD1) were treated with 50 nM DOX and 50 nM PTX that served as positive controls for the induction of P-gp expression. Then, cells were collected by trypsinization, washed with PBS, and directly immunostained by FITC-conjugated anti-P-gp antibody according to the manufacturer’s protocol. The samples were kept in the dark until the analysis on CyFlow Space Partec flow cytometer (Sysmex Partec GmbH, Görlitz, Germany). The fluorescence of FITC-conjugated anti-P-gp was detected on fluorescence channel 1 (FL1-H) at 530 nm. A minimum of 10,000 events were collected for each sample (the gate excluded cell debris and dead cells) and the obtained results were analyzed with Summit software v4.3 (Dako Colorado Inc., Fort Collins, CO, USA).

### 3.9. Rhodamine 123 Accumulation Assay

The accumulation of Rho 123, a fluorescent P-gp substrate, was measured by flow cytometry. The intensity of the fluorescence is proportional to Rho 123 accumulation in the cell. All cell lines were seeded in adherent 6-well plates and grown overnight. DLD1-TxR and NCI-H460/R cells were treated for 30 min with the following concentrations of Hsp90 inhibitors: 500 nM compound 3, 1 µM compound 4, 10 µM compound 5, 500 nM compound 6, 500 nM compound 7, 1 µM compound 8, 500 nM compound 9, 1 µM compound 10, 1 µM compound 13, 10 µM compound 14, 100 nM compound 15. TQ (10 nM) was used as a positive control for Rho 123 accumulation. Rho 123 (5 μM) was simultaneously added with the investigated compounds and the cells were incubated at 37 °C in 5% CO_2_ for 30 min. The cells were then trypsinized, pelleted by centrifugation, washed with PBS and placed in ice-cold PBS. The samples were kept on ice in the dark until the analysis on CyFlow Space Partec flow cytometer (Sysmex Partec GmbH, Görlitz, Germany). The fluorescence of Rho 123 was detected on fluorescence channel 1 (FL1-H) at 530 nm. A minimum of 10,000 events were collected for each sample and the obtained results were analyzed using Summit software.

### 3.10. P-gp ATPase Activity Assay

P-gp relies on the ATP hydrolysis energy to extrude the substrate outside the cell and compounds that interact with P-gp can stimulate or inhibit its ATPase activity. Compounds that are P-gp substrates usually stimulate its ATPase activity [53]. Pgp-Glo™ assay system (Promega, Madison, WI, USA) detects the compound’s effect on recombinant human P-gp in a cell membrane fraction. P-gp ATPase activity was measured using luminescent Pgp-Glo™ assay kit according to manufacturer’s instructions. Briefly, ATP was first incubated with P-gp; then the P-gp ATPase reaction was stopped, and the remaining unmetabolized ATP was detected as a luciferase-generated luminescent signal. Pgp-dependent decreases in luminescence reflect ATP consumption by P-gp; thus, the greater the decrease in signal, the higher the P-gp activity. Accordingly, samples containing compounds that stimulate the P-gp ATPase will have significantly lower signals than untreated samples. Verapamil and sodium orthovanadate, supplied in the assay kit were used as positive controls. Verapamil is a P-gp substrate and competitive inhibitor, and sodium orthovanadate is a noncompetitive inhibitor of P-gp ATPase activity. The luminescence of samples treated with increasing concentrations of compound 5 (5 µM, 10 µM and 20 µM), compound 7 (100 nM, 250 nM and 500 nM) and compound 9 (100 nM, 250 nM and 500 nM), was measured with a luminometer microplate reader (CHAMELEON™V, Hidex, Turku, Finland).

### 3.11. Cell Death Analysis

Induction of cell death after Hsp90 inhibitors treatment was assessed by dual staining with Annexin V—FITC/Propidium Iodade (AV/PI). NCI-H460, DLD1 and MRC-5 cells were seeded in adherent 6-well plates and incubated overnight. All cell lines were treated with 5 µM compound 5, 200 nM compound 7, and 100 nM compound 9. After 72 h, both attached and floating cells were collected and re-suspended in 50 mL of binding buffer, containing AV and PI in ratio 1:1 (*v*/*v*). After 10 min at room temperature in the dark, an additional 200 mL of binding buffer was added, and AV/PI staining was analyzed within 1 h by flow cytometry. The fluorescence intensities of AV and PI were measured in green FL1 and red FL2 channel, respectively, on CyFlow Space flow cytometer (Sysmex Partec GmbH, Görlitz, Germany). In each sample, the acquisition of 20,000 cells was performed, and the percentages of viable (AV−PI−), early apoptotic (AV+PI−), late apoptotic (AV+PI+), and necrotic (AV−PI+) cells were analyzed by Summit software.

### 3.12. Median Effect Analysis

The combined effects of compound 5 with DOX as well as with PTX were studied in NCI-H460/R and DLD1-TxR cells, respectively, by MTT assay. In simultaneous treatments that lasted 72 h in NCI-H460/R cells, three concentrations of compound 5 (2.5, 5, and 10 µM) were combined with increasing concentrations of DOX (250, 500 and 1000 nM), while in DLD1-TxR cells different concentrations of compound 5 (1, 2.5, and 5 µM) were combined with increasing concentrations of PTX (100, 250 and 500 nM). The nature of the interaction between Hsp90 inhibitors and DOX/PTX was analyzed with CalcuSyn software v1.1 (Biosoft, Cambridge, UK) that uses the Combination Index method based on the multiple drug effect equation [54]. Three data points were used for each single drug in each experiment. The non-constant ratio combination was chosen to assess the effect of both drugs in combination. The results are presented in a fraction-affected CI graph. Values of CI < 1 point to a pronounced additive effect or synergism (the smaller value, the greater the degree of synergy). A value of CI = 1 indicates an additive effect, while values of CI > 1 point to an antagonistic effect.

### 3.13. In Silico Studies

For the docking simulations MOE v. 2019.01 software was applied (MOE, Molecular Operating Environment, Chemical Computing Group Inc., Montreal, QC, Canada).

Preparation of the structures was as follows: The structures of TQ, compounds 5, 7 and 9 were built and geometrically optimized using MMFF94s force field in MOE. Prior to docking, the P-gp structures were processed in the “Protonate 3D” tool of MOE. This tool prepares the protein structure by (I) assigning hydrogens to structures following the optimal free energy proton geometry; (ii) assigning the ionization states of titratable protein groups using the Generalized Born electrostatics model. The following settings were used to mimic physiologically relevant conditions: temperature of 300K; pH = 7.4; ion concentration of 0.1 mol/L.

Docking: In the docking protocol the “Triangle Matcher” placement method was used and the poses were ranked using London dG scoring function. Further refinement was performed using “Rigid receptor” and final scoring was based on London dG scoring function.

Ligand-receptor interactions: This analysis was performed in the “Ligand interactions” tool of MOE for the selected binding poses. The algorithm ascertains the interactions between the ligand and the species immediately surrounding it, and decides which residues, solvents and ions are to be included in the resulting output.

### 3.14. Statistical Methods

Statistical significance for the flow cytometric data on Rho 123 accumulation was calculated by two-way ANOVA using Sidak’s multiple comparisons test. Statistical significance for the flow cytometric data on P-gp expression was determined with unpaired Student’s *t*-test with Welch’s correction. Statistical analysis for combination treatments was performed by a two-way ANOVA test. P-gp ATPase activity assay data were analyzed by unpaired Student’s t-test. Statistical significance was set at *p* < 0.05. All statistical analysis was performed in GraphPad Prism 6.0 software (San Diego, CA, USA).

## 4. Conclusions

Recognizing the potential of compounds to inhibit function and/or expression of P-gp is vital in the search for efficient strategies for combating resistant cancers. In addition, the development of dual-targeting inhibitors with capability to overcome MDR could lead to better efficacy, selectivity and tolerability and better prospects for effective cancer treatment. In this study, we evaluated the potential of eleven novel Hsp90 inhibitors to influence the P-gp activity and expression and evade the MDR phenotype in two resistant cancer cell lines with P-gp overexpression. Three investigated compounds displayed strong P-gp-modulating potential and emerged as new promising dual Hsp90 and P-gp inhibitors. Thus, we propose these compounds could be considered as lead candidates for developing new compounds with improved characteristics for dual Hsp90 and P-gp targeting.

## Figures and Tables

**Table 1:** Cell growth inhibition (presented as IC_50_ values in μM) induced by Hsp90 inhibitors with different affinity of binding to Hsp90.

Compound	NCI-H460	NCI-H460/R	DLD1	DLD1-TxR	MRC-5 ^#^	Relative Selectivity ^RS^	Hsp90 Affinity binding (μM) [15]	LogP
3	0.785 ± 0.032 *	0.599 ± 0.020 ^CS^	0.925 ± 0.032	0.644 ± 0.020 ^CS^	0.636 ± 0.023	NO	7.850	3.24
4	0.935 ± 0.048	0.579 ± 0.018 ^CS^	1.523 ± 0.054	1.059 ± 0.025 ^CS^	1.747 ± 0.083	1.71	5.840	3.08
5	5.722 ± 0.073	25.496 ± 0.925 ^R^	5.683 ± 0.163	11.060 ± 0.304 ^R^	127.677 ± 38.976	10.64	1.550	2.93
6	0.807 ± 0.035	0.472 ± 0.018 ^CS^	0.439 ± 0.006	0.439 ± 0.010 ^E^	4.737 ± 0.473	8.79	1.200	2.88
7	0.199 ± 0.005	0.231 ± 0.006 ^E^	0.156 ± 0.004	0.138 ± 0.003 ^E^	0.599 ± 0.015	3.31	0.390	2.02
8	0.159 ± 0.003	0.351 ± 0.005 ^R^	0.251 ± 0.003	3.364 ± 0.049 ^R^	11.021 ± 0.366	10.69	0.034	-0.20
9	0.096 ± 0.002	0.172 ± 0.005 ^R^	0.045 ± 0.001	0.050 ± 0.001 ^E^	0.302 ± 0.008	3.33	0.540	2.02
10	1.269 ± 0.014	1.375 ± 0.012 ^E^	2.645 ± 0.035	1.787 ± 0.036 ^CS^	0.257 ± 0.004	NO	1.060	2.40
13	1.250 ± 0.028	1.060 ± 0.012 ^E^	1.895 ± 0.036	1.004 ± 0.016 ^CS^	0.174 ± 0.004	NO	1.900	1.86
14	0.978 ± 0.018	23.597 ± 0.470 ^R^	7.449 ± 0.212	10.632 ± 0.270 ^R^	53.283 ± 1.729	5.00	0.340	–0.36
15	0.052 ± 0.002	0.039 ± 0.001 ^E^	0.044 ± 0.001	0.193 ± 0.007 ^R^	0.250 ± 0.014	3.05	0.084	1.63

* All values are presented as an average IC_50_ from three independent experiments and given in μM. ^#^ MRC-5 human embryonic fibroblasts were used to determine relative selectivity to cancer cells. ^CS^ Collateral Sensitivity (IC_50_ values lower in MDR cells than in corresponding sensitive cells). ^R^ Resistance (IC_50_ values higher in MDR cells than in corresponding sensitive cells). ^E^ Evading resistance mechanisms (similar IC_50_ values between MDR and corresponding sensitive cells). ^RS^ Relative selectivity towards cancer cells: A relation between IC_50_ value obtained in MRC-5 cells and an average IC_50_ value from four tested cancer cell lines.

**Table 2:** mRNA HSP90 relative expression in NCI-H460, NCI-H460/R, DLD1 and DLD1-TxR cells.

Cell Lines	NCI-H460	NCI-H460/R	DLD1	DLD1-TxR
Relative mRNA *HSP90* expression	1.000 ± 0.001	0.240 ± 0.034 *	0.477 ± 0.018 ^#^	0.356 ± 0.016 ^#^

* significant difference compared to corresponding sensitive cell line; ^#^ mRNA *HSP90* expression relative to NCI-H460 cells; results are expressed as mean ± SD of three replicates.

**Table 3:** Relative reversal of doxorubicin (DOX) and paclitaxel (PTX) resistance in NCI-H460/R and DLD1-TxR cells induced by Hsp90 inhibitor.

Compound	DOX IC_50_ (nM)	Relative Reversal
**DOX**	2265.6 ± 42.4	
5		
2.5 µM	1731.4 ± 30.9	1.31
5 µM	863.9 ± 25.6	2.62
10 µM	429 ± 13.3	5.28
**PTX**	1528.5 ± 48.6	
5		
1 µM	850.4 ± 13.1	1.80
2.5 µM	588.3 ± 12.0	2.60
5 µM	290.0 ± 5.0	5.27

## Abbreviations

HSPs	Heat shock proteins
ABC	ATP Binding Cassette
MDR	multidrug resistance
P-gp	P-glycoprotein
DOX	doxorubicin
PTX	paclitaxel
Rho 123	rhodamine 123
TQ	tariquidar
CI	Combination Index
DMSO	dimethyl sulfoxide
FBS	fetal bovine serum
MTT	thiazolyl blue tetrazolium bromide
